# Ovarian Reserve After Robotic Versus Laparoscopic Single-Site Ovarian Cystectomy for Mature Cystic Teratoma: A Prospective Comparative Study

**DOI:** 10.3390/jcm14113800

**Published:** 2025-05-29

**Authors:** Seyeon Won, Su Hyeon Choi, Nara Lee, So Hyun Shim, Mi Kyoung Kim, Mi-La Kim, Yong Wook Jung, Jin Young Paek, Seok Ju Seong

**Affiliations:** 1 Department of Obstetrics and Gynecology, CHA Gangnam Medical Center, CHA University School of Medicine, 566, Nonhyeon-ro, Gangnam-gu, Seoul 06135, Republic of Korea; djtong85@naver.com (S.W.); k345@chamc.co.kr (S.H.C.); naradd@chamc.co.kr (N.L.); simuso@chamc.co.kr (S.H.S.); ra13811@chamc.co.kr (M.K.K.); mila76@cha.ac.kr (M.-L.K.); dumbung@chamc.co.kr (Y.W.J.); 2 Department of Laboratory Medicine, CHA Gangnam Medical Center, CHA University School of Medicine, 566, Nonhyeon-ro, Gangnam-gu, Seoul 06135, Republic of Korea

**Keywords:** robotic surgical procedures, laparoscopy, ovarian surgery

## Abstract

**Objectives**: This study aimed to compare the impact of robotic (RO) versus laparoscopic single-site ovarian cystectomy (LO) on ovarian reserve, as measured by changes in anti-Müllerian hormone (AMH) levels. **Methods**: A total of 43 women undergoing LO and 40 women undergoing RO for mature cystic teratoma were included. The baseline characteristics and surgical outcomes were scrutinized. AMH levels were evaluated preoperatively and at 3 months after surgery. **Results**: BMI (body mass index) was higher in the LO group (23.1 ± 3.6 cm vs. 21.7 ± 2.1 kg/m^2^, *p* = 0.03) than in the RO group. Otherwise, there were no statistically significant differences in patient characteristics. The LO group showed a shorter operative time (70.0 ± 24.0 vs. 86.5 ± 26.7 min, *p* = 0.002) than the RO group., However, multiple linear regression analysis revealed that BMI was independently associated with increased operative time (*p* = 0.001), while the surgical method was not a significant predictor (*p* = 0.725). There were no significant differences in the rate of decline in AMH level between the LO and RO groups (13.3 ± 21.2 vs. 4.64 ± 34.1%, *p* = 0.167). There were intergroup differences in the hemostasis method: in the LO group, bipolar coagulation was performed for 27 (62.8%) patients, and sutures were performed for 16 (37.2%) patients; in the RO group, bipolar coagulation was performed for 5 (12.5%) patients, and sutures were performed for 33 (82.5%) patients, while in the case of 2 (5.0%) patients, hemostasis was not performed (*p* < 0.001). However, note that in our logistic regression analysis, the hemostasis method was excluded as an independent factor affecting AMH. **Conclusions**: There appear to be no significant advantages of RO over LO in terms of ovarian reserve preservation.

## 1. Introduction

Since its introduction, the application of Da Vinci robotic surgery has been gradually expanding in the fields of gynecology and general surgery [1]. Da Vinci robotic surgery offers a 3D view and enhances maneuverability with favorable ergonomics, along with the additional remarkable advantage of making the most challenging process in laparoscopy, suturing, much easier [2].

However, studies proving that robotic surgery significantly outperforms conventional laparoscopy in terms of surgical outcomes are still difficult to find. A Cochrane review analyzing 12 randomized controlled trials (RCTs) on robotic gynecological surgery indicated that whereas robotic hysterectomy relative to laparoscopic hysterectomy may require more surgical time, there were no significant differences in other outcomes [3]. Prospective studies on myomectomy or ovarian cystectomy outcomes of robotic surgery also are scarce, and although there are several retrospective comparisons regarding the surgical efficacy for myomectomy or ovarian surgery, the results are mixed [4,5,6]. Although several retrospective studies have attempted to compare robotic and laparoscopic approaches, inherent biases and heterogeneity in surgical indication, patient selection, and perioperative management limit the strength of their conclusions. Thus, prospective, controlled studies remain essential to provide more definitive evidence.

Within gynecologic surgery, procedures involving the ovary present distinct clinical challenges compared to those targeting the uterus. In particular, ovarian-preserving surgeries for benign ovarian cysts demand careful consideration of the surgical technique to minimize trauma and preserve hormonal function. Factors such as cyst type, tissue characteristics, and the risk of thermal or mechanical injury can influence postoperative ovarian reserve. These variations emphasize the importance of assessing surgical platforms in the context of specific anatomical and functional goals rather than extrapolating findings from other gynecologic procedures.

Preserving ovarian function is a crucial aspect for young women undergoing ovarian surgery. Various comparative studies, accordingly, have scrutinized surgical methods for their effectiveness in preserving ovarian function.

Robotic surgery, due to its technical precision and improved dexterity, has been hypothesized to offer potential advantages in minimizing intraoperative damage to the ovary. Further investigation is warranted to clarify whether these theoretical advantages translate into measurable clinical benefits. This study aimed to evaluate the effect of different surgical methods on ovarian function following ovarian cystectomy.

## 2. Materials and Methods

### 2.1. Study Design

**Figure 1:**
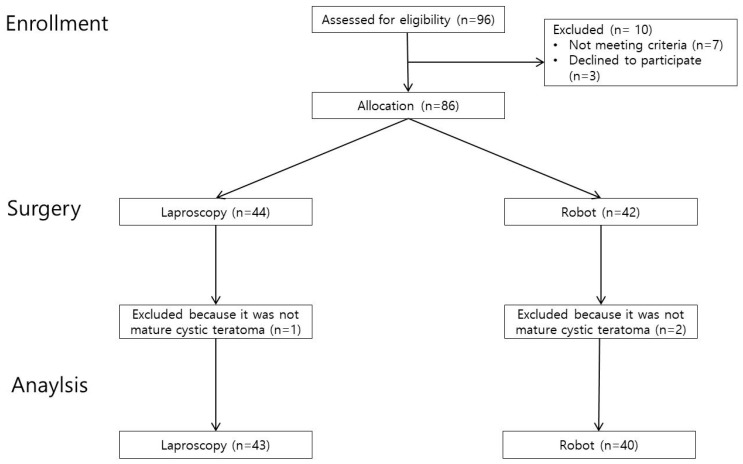
Flow diagram of patient selection.

This study recruited patients who underwent RO or LO between August 2020 and June 2023 for suspicion of mature cystic teratoma. The primary objective of this study was to compare the impact of robotic versus laparoscopic single-site ovarian cystectomy on ovarian reserve, as assessed by changes in anti-Müllerian hormone (AMH) levels. The inclusion criteria were as follows: (1) cyst size between 3 and 10 cm, (2) age between 21 and 40 years, and (3) regular menstrual cycle (21–35 days). The exclusion criteria were as follows: (1) previous history of ovarian cyst surgery, (2) suspicious findings regarding malignant ovarian diseases, (3) use of sex-hormone-related medications (e.g., oral contraceptives, GnRH agonists, estrogen, or progestin) within 3 months before or after surgery, and (4) Postoperaive histological reports indicated a diagnosis other than mature cystic teratoma. A total of 96 patients were initially screened for eligibility during the study period. Of these, 7 did not meet the inclusion criteria and 3 withdrew consent. After further exclusion based on final pathological diagnosis, 83 patients were included in the final analysis—43 in the LO group and 40 in the robotic RO group. The patient selection process is illustrated in Figure 1.

### 2.2. Surgical Procedures

#### 2.2.1. Robotic Single-Site Ovarian Cystectomy (RO)

Initially, a single-site port of entry was created through a 2.0 cm incision at the umbilicus using a wound retractor. Following this, a Glove port (Nelis, Seoul, Republic of Korea) was inserted. Subsequently, the robotic system (Si or Xi) was vertically docked, and an 8.5 mm 30° Da Vinci stereo laparoscope was introduced. After docking, a semi-rigid robotic instrument such as a monopolar hook, along with a bipolar forceps, was placed in the robotic arms. An incision at the ovary was made using the monopolar hook, and the cyst was enucleated. Bleeding was controlled for hemostasis, either by bipolar coagulation or suturing with barbed sutures using a robotic needle holder. In cases of mild bleeding, hemostatic agents such as Surgicel^®^ (Ethicon, Somerville, NJ, USA) were used without other coagulation methods. The cyst was retrieved through the umbilical incision. The peritoneum, fascia, and subcutaneous layers were closed using 1-0 vicryl suture materials (Ethicon, Somerville, NJ, USA), and the skin was closed using 3-0 vicryl suture materials.

#### 2.2.2. Laparoscopic Single-Site Ovarian Cystectomy (LO)

The surgery was performed using the same techniques as in the robotic surgery, except that instead of robotic instruments, a 30-degree laparoscopic camera, laparoscopic bipolar and monopolar instruments, and a needle holder were employed.

### 2.3. Data Analysis

For each patient, the following variables were recorded: age, body mass index (BMI), ovarian cyst size, laterality of the lesion, operative time, estimated blood loss, hospital stay duration, hemoglobin drop, preoperative and postoperative AMH levels, surgical complications, and method of hemostasis. The primary outcome was the extent of AMH reduction before and 3 months after surgery. This was calculated using the following formula: rate of decline in AMH levels (%) = 100 × (preoperative AMH − postoperative AMH)/preoperative AMH. Blood samples were collected from patients to assess the serum AMH level. After separation from whole blood, the serum was transferred to sterile polypropylene tubes and stored at −70 °C. Serum AMH concentrations were determined using an enzyme immunoassay kit following the manufacturer’s instructions (Immunotech version, Beckman Coulter, Marseille, France). The assay’s detection limit for AMH was 0.14 ng/mL, with intra- and inter-assay coefficients of variation for the AMH assay being below 12.3 and 14.2%, respectively. Secondary outcomes included operative time, estimated blood loss, hemoglobin drop, hospital stay duration, and postoperative complications. The operating time was defined as the duration from skin incision to skin closure. Hemoglobin (Hb) drop was characterized by the decrease in Hb levels from preoperative values to day 1 post-surgery.

### 2.4. Statistical Analysis

Since there were no previous studies of the same nature, we referenced the data from Wang et al. [7] to calculate the sample size. In Wang et al.’s study comparing the extent of AMH reduction after single-port laparoscopic ovarian cystectomy and conventional laparoscopic ovarian cystectomy, the mean AMH reduction difference was 1.13 ng/mL, with a standard deviation of 1.39. Using this information and aiming for a statistical power of 95% at an alpha of 0.05, it was determined that 82 patients, with 41 in each group, were needed. Accounting for an anticipated dropout rate of 12%, the final decision was to recruit a total of 92 patients, with 46 in each group. Baseline characteristics were summarized using means and standard deviations for continuous variables and counts and percentages for categorical variables. Between-group comparisons were conducted using Student’s *t*-test for continuous variables and the chi-square test or Fisher’s exact test for categorical variables, as appropriate. The primary outcome—the percentage change in AMH levels before and 3 months after surgery—was compared between groups using the independent *t*-test. Surgical outcomes, including operative time, blood loss, hospital stay, and complication rates, were analyzed using the same tests depending on the data type. To assess independent predictors of operative time, a multiple linear regression analysis was performed using BMI and surgical method (robotic vs. laparoscopic) as covariates. Additionally, variables with a *p*-value < 0.2 in the univariate analysis were entered into a multivariate logistic regression model to evaluate factors associated with a significant AMH decline.

All of the analyses were performed using SPSS version 24.0 (IBM Inc., Armonk, NY, USA), with *p*-values < 0.05 considered statistically significant.

### 2.5. Ethical Approval and Trical Registration

This prospective study received IRB approval from Cha Gangnam Medical Center (GCI 2019-11-049-029). Written informed consent was obtained from all participants prior to study enrollment. This study was prospectively registered in the CRIS database (KCT0005236) on 16 July 2020.

## 3. Results

**Figure 2:**
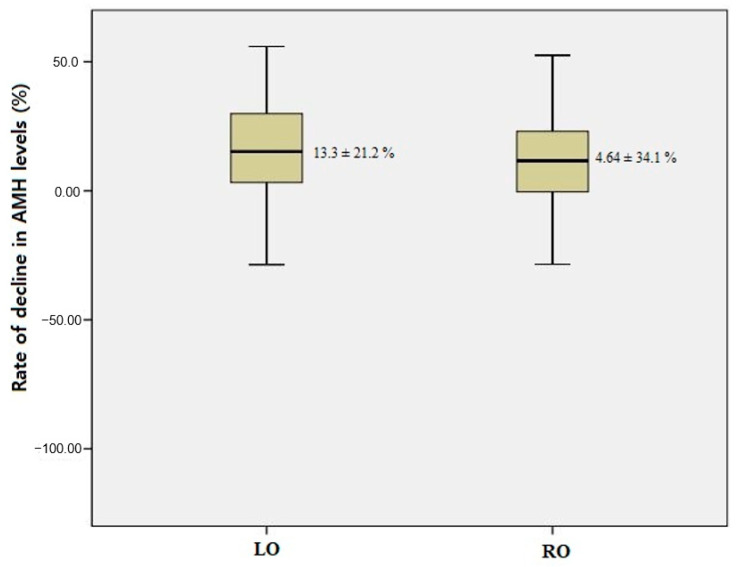
Rate of decline in AMH levels between surgical platforms.

A total of 83 patients were included in the final analysis, with 43 in the laparoscopic (LO) group and 40 in the robotic (RO) group. The LO group exhibited a higher BMI (23.1 ± 3.6 vs. 21.7 ± 2.1, *p* = 0.030) relative to the RO group. However, no significant intergroup differences were observed in any other characteristics, including age and ovarian cyst size. Specifically, the mean ovarian cyst size was 6.7 ± 2.2 cm in the LO group and 7.1 ± 1.7 cm in the RO group (*p* = 0.354), confirming comparability between the groups in this regard. These findings are summarized in Table 1. In terms of surgical outcomes, the LO group demonstrated a significantly shorter operative time compared to the RO group (70.0 ± 24.0 vs. 86.5 ± 26.7 min, *p* = 0.002). Estimated blood loss and hemoglobin decrement were similar between the groups, with no significant differences (*p* = 0.673 and *p* = 0.675, respectively). Concurrent surgeries, including myomectomy, paratubal cystectomy, and hysteroscopy, were infrequently performed, and the rates did not significantly differ between the groups (*p* = 0.100). Hospital stay duration was comparable (4.1 ± 0.7 days in the LO group vs. 4.0 ± 0 days in the RO group, *p* = 0.070). No major intraoperative complications or conversions were reported. Postoperative complications were minimal, with two cases (4.7%) of postoperative ileus observed in the LO group and none in the RO group (*p* = 0.495). These surgical outcomes are presented in Table 2. These results are presented in Table 2. To further investigate the factors influencing operative time, a multiple linear regression analysis was conducted, including BMI and surgical method (RO vs. LO) as independent variables. The analysis revealed that BMI was significantly associated with operative time (B = 3.242, 95% confidence interval [CI]: 1.284–5.201, *p* = 0.001), indicating that for each one-unit increase in BMI (kg/m^2^), the operation time increased by approximately 3.2 min on average. Conversely, the surgical method itself was not a statistically significant predictor of operative time (*p* = 0.725), suggesting that the initially observed longer operative time in the RO group may have been confounded by differences in patient BMI. The overall model was statistically significant (*p* = 0.009), with an adjusted R^2^ of 0.088, indicating that BMI and surgical method together accounted for approximately 8.8% of the variability in operative time. Regarding the hemostasis methods, a significant difference (*p* < 0.001) was observed. In the LO group, 62.8% of patients underwent bipolar coagulation, and 37.2% received sutures for hemostasis. By contrast, in the RO group, 5.0% relied solely on surgical hemostatic sponges, 12.5% underwent bipolar coagulation, and for 82.5%, sutures were used for hemostasis. There were no significant differences in the preoperative AMH levels between the LO and RO groups (4.26 ± 2.04 vs. 4.09 ± 1.66, *p* = 0.677) nor in the AMH levels 3 months post-surgery (3.75 ± 1.91 vs. 3.90 ± 1.70, *p* = 0.720). The rate of decline in AMH levels did not differ significantly between the two groups (*p* = 0.167), as depicted in Figure 2. Univariate and multivariate analyses were performed to identify factors associated with the rate of decline in AMH levels. Surgical method (RO vs. LO), BMI, hemostasis method, and operative time were not found to be independent predictors. These results are summarized in Table 3.

## 4. Discussion

In summary of this study’s findings, there was no difference between the two platforms regarding their impact on ovarian function. Three retrospective studies have been published comparing the extent of AMH level decline between RO and LO. However, the results and study designs varied, making it difficult to draw meaningful conclusions. In Lee’s published study [6], the outcomes for 40 patients who underwent RO and 54 patients who underwent LO for endometrioma were retrospectively compared. Only in the bilateral endometrioma cases was a lower decline in AMH level reported for the RO group. Kang et al. published the results of a similar study [4] that included 87 patients who underwent RO and 78 patients who underwent LO for endometrioma. They reported that in cases of mild or non-complex endometriosis, the extent of AMH decline was lower with RO, and overall, there was no difference in the extent of AMH decline. In the study by Lee et al. [5], other benign cysts were also included in their analyses. Having compared the outcomes for 84 patients who underwent RO with those for 72 patients who underwent LO, they reported a lower AMH decline with RO. Such disparate results, in our opinion, were due to endometrioma, the targeted condition in that study, which requires postoperative hormone therapy. According to the 2022 ESHRE Guidelines, long-term hormone treatment is recommended for women who do not seek conception after surgery [8]. Generally, the use of GnRH agonists, dienogest, or oral contraceptives and other hormonal treatments is common. However, it is challenging to adjust for their individual impacts on AMH levels. Nonetheless, there are various reports suggesting that different hormonal therapies influence AMH levels variously [9,10,11]. Consequently, we planned a study targeting mature cystic teratoma, where postoperative hormonal therapy is not required, so as to thoroughly evaluate the impact of surgery on AMH levels.

Contrary to our presumption that RO would have advantages, it was confirmed that there was no difference in the extent of AMH decline. While there may indeed be no real advantage to RO, we speculate that our result could be attributed to the lesser impairment of ovarian function during surgery for mature cystic teratoma relative to other ovarian disorders such as endometrioma. According to Mansouri et al. [12], cyst type was a contributing factor to AMH level decrease, with the lowest decrease reported for mature cystic teratoma relative to endometrioma and cystadenoma (*p* ≤ 0.001). Ergun et al. [13] and Hartono et al. [14] discovered that endometriosis, which involves various mechanisms such as inflammation and heightened oxidative stress within the tissue, leads to a greater reduction in serum AMH levels compared with other ovarian cysts. We have considered the possibility that if we had conducted a study with a design that uniformly adjusted for postoperative hormonal therapy in endometrioma cases, we might have obtained different results. The impacts of different hemostatic methods during surgery may vary depending on ovarian function. According to Baracat et al.’s meta-analysis [15], suturing is to be recommended over bipolar coagulation, as it results in a lower extent of AMH decline. In our study, despite a significantly higher proportion of cases using suturing during RO (82.5 vs. 37.2%, *p* < 0.001), there was no difference in the extent of AMH decline. Additionally, operation time was longer in RO (70.0 ± 24.0 vs. 86.5 ± 26.7, *p* = 0.002). However, multiple linear regression analysis revealed that BMI was independently associated with increased operative time, while the surgical method itself was not statistically significant. This suggests that the prolonged operative time observed in the RO group may be attributed to higher BMI rather than to the surgical technique itself. This finding is consistent with previous reports indicating that a higher BMI is associated with longer operative times in laparoscopic surgeries [16,17,18]. Several studies have demonstrated that increased BMI can complicate surgical exposure, instrument maneuverability, and operative field visualization, thereby prolonging the duration of minimally invasive procedures. Our results align with these findings, suggesting that BMI is a crucial patient-related factor influencing operative duration, even when advanced platforms like robotic surgery are utilized. These observations underscore the importance of considering patient BMI in preoperative planning and surgical approach selection. Considering the comparable outcomes in ovarian reserve preservation, LO may be regarded as a more efficient and practical approach for treating mature cystic teratoma in appropriately selected cases.

Although the present study was prospective in design, the characteristics of the two groups were not identical. Differences in BMI and hemostasis method could be considered to be limitations. Although there was no statistically significant difference in cyst size between the laparoscopic and robotic groups in this study, ovarian cyst size itself may affect postoperative ovarian reserve. Therefore, future studies may benefit from stratifying patients by cyst size to better assess its potential impact on ovarian function. Another limitation is the inability to evaluate other benign cysts. In fact, for patients with endometriosis, fertility issues are often a concern, making endometriosis perhaps the most suitable condition for evaluating the extent of ovarian function decline. Although we did not obtain significant results for mature cystic teratoma, conducting a prospective study targeting endometriosis may yield different results. Finally, the transition from the XI/SI system to the SP system in RO is ongoing, and our inability to evaluate the new SP system is another limitation of our study. However, being the first prospective study to evaluate the impact of RO on ovarian function is a significant strength of the present research.

In clinical practice, the preservation of ovarian reserve is a key consideration for reproductive-aged women undergoing surgery for benign ovarian diseases. When selecting a surgical method, it is crucial not only to assess technical feasibility but also to consider the potential impact on reproductive function. Our findings indicate that for mature cystic teratoma, RO does not offer a clear advantage over LO in terms of ovarian function preservation. Although the operative time appeared shorter in the laparoscopic group, multiple linear regression analysis revealed that the surgical method itself was not independently associated with operative time. Given these results, surgical method selection for mature cystic teratoma should prioritize ovarian function preservation, patient characteristics, and surgeon expertise rather than focusing solely on operative duration. While robotic surgery provides technical benefits such as enhanced visualization and instrument dexterity, it also entails substantially higher costs compared to conventional laparoscopy, including equipment acquisition, maintenance, and longer operating times. In the absence of demonstrated superiority in ovarian function preservation, the routine application of robotic platforms for benign ovarian diseases like mature cystic teratoma should be approached cautiously. Particularly in healthcare systems where the sustainability of medical resources is a pressing concern, cost-effectiveness must be carefully considered when making surgical decisions. Future research, including large-scale prospective studies and formal economic evaluations, is warranted to establish more definitive guidelines for the optimal surgical approach.

## 5. Conclusions

There appear to be no significant advantages of RO over LO in terms of ovarian reserve preservation. Further and larger prospective cohort studies are needed to validate the effects of RO on ovarian reserve, especially in cases of endometrioma. 

## Figures and Tables

**Table 1:** Baseline characteristics in women who underwent LO and RO.

Characteristics	LO (n = 43)	RO (n = 40)	*p*
Age, year	30.4 ± 4.9	29.7 ± 4.9	0.490
BMI, kg/m^2^	23.1 ± 3.6	21.7 ± 2.1	0.030
Married			0.951
No	32 (74.4)	30 (75.0)	
Yes	11 (25.6)	10 (25.0)	
Nulliparous			0.316
No	36 (83.7)	37 (92.5)	
Yes	7 (16.3)	33 (72.5)	
Previous abdominal surgery			0.435
No	38 (88.4)	38 (95.0)	
Yes	5 (11.6)	2 (5.0)	
Peritoneal adhesion			0.111
No	6 (14.0)	1 (2.5)	
Yes	37 (86.0)	39 (97.5)	
Diameter of ovarian cyst, cm	6.7 ± 2.2	7.1 ± 1.7	0.354
Location of ovarian cyst			0.677
Unilateral	39 (90.7)	38 (95.0)	
Bilateral	4 (9.3)	2 (5.0)	

Values are presented as numbers (%), medians (range), or mean ± standard deviations. RO, robotic single-site ovarian cystectomy; LO, laparoscopic single-site ovarian cystectomy; BMI, body mass index.

**Table 2:** Surgical characteristics and outcomes.

Characteristics	LO (n = 43)	RO (n = 40)	*p*
Operation time, min	70.0 ± 24.0	86.5 ± 26.7	0.002
Estimated blood loss, mL	64.2 ± 37.2	61.0 ± 30.8	0.673
Hemoglobin decrement, g/dL	2.0 ± 0.7	2.0 ± 0.8	0.675
Concurrent surgery			0.100
No	38 (90.5)	31 (77.5)	
Myomectomy	1 (2.4)	1 (2.5)	
Paratubal cystectomy	1 (2.4)	3 (7.5)	
Hysteroscopy	2 (4.8)	5 (12.5)	
Hospital stay, days	4.1 ± 0.7	4.0 ± 0	0.070
Hemostasis methods			<0.001
None	0 (0)	2 (5.0)	
Bipolar coagulation	27 (62.8)	5 (12.5)	
Suture	16 (37.2)	33 (82.5)	
Complications			0.495
None	41 (95.3)	40 (100)	
Reoperation within 1 week	0 (0)	0 (0)	
Ileus	2 (4.7)	0 (0)	
Fever > 3 days	0 (0)	0 (0)	
Ovarian reserve			
Preoperative AMH level, ng/mL	4.26 ± 2.04	4.09 ± 1.66	0.677
Postoperative AMH level, ng/mL	3.75 ± 1.91	3.90 ± 1.70	0.720
Rate of decline in AMH level, %	13.3 ± 21.2	4.64 ± 34.1	0.167

Values are presented as numbers (%), medians (range), or mean ± standard deviations. RO, robotic single-site ovarian cystectomy; LO, laparoscopic single-site ovarian cystectomy; AMH, anti-Müllerian hormone.

**Table 3:** Risk factors affecting the rate of AMH decline.

	Univariate Analysis		Multivariate Analysis	
Clinical Factors	OR(95% CI)	*p*	OR(95% CI)	*p*
Rate AMH decline ≥ 14.0 ^a^				
RO (vs. LO)	1.107(0.383–3.198)	0.851		
BMI ≥ 21.9 kg/m^2 a^ (vs. <21.9 kg/m^2^)	1.107(0.383–3.198)	0.184	0.547(0.218–1.373)	0.199
Suture (vs. bipolar coagulation)	0.422(0.137–1.300)	0.133	2.061(0.803–5.286)	0.132
OP time ≥ 70 min ^a^ (vs. <70 min)	1.301(0.498–3.400)	0.591		

AMH, anti-Müllerian hormone; CI, confidence interval; OR, odds ratio; BMI, body mass index; RO, robotic single-site ovarian cystectomy; LO, laparoscopic single-site ovarian cystectomy; OP, operation; ^a^ median value in study population.

## Data Availability

The data presented in this study are available on request from the corresponding author.
